# Thymoma with Coexisting Undifferentiated Pleomorphic Sarcoma: A Case Report

**DOI:** 10.1155/2011/135794

**Published:** 2011-10-29

**Authors:** Manoranjan Varshney, Mohammad Shahid, Veena Maheshwari, Aysha Mubeen, Mohammed Azfar Siddiqui

**Affiliations:** Department of Pathology, J. N. Medical College, AMU, Aligarh, India

## Abstract

We report here a case of thymoma simultaneously associated with undifferentiated pleomorphic sarcoma. A 45-year-old male presented with axillary lump. Radiographic studies showed a mediastinal mass. On fine needle aspiration cytology and histopathological examination, a diagnosis of thymoma with coexisting undifferentiated pleomorphic sarcoma was made. Although thymomas are associated with many extrathymic malignancies, it's association with undifferentiated pleomorphic sarcoma is rare. This case is being reported on to reinforce that clinicians should bear in mind the possibility of extrathymic malignancies in patients with thymomas.

## 1. Introduction

Thymoma is a tumor of anterior mediastinum, however, thymomas may develop at various other sites like neck, trachea, thyroid, lung, and heart [1]. It is the most common neoplasm of thymus, more common in men and in eighth decade of life [2]. Thymoma is an uncommon neoplasm which arises from thymic epithelium. Thymomas are associated with paraneoplastic syndromes such as myasthenia gravis, hypogammaglobulinemia [3], pure red cell aplasia [4], and many kinds of immune mediated systemic disorders; however, few cases of extrathymic malignancies have been reported. Reported incidence of extrathymic malignancies is 2.6%–27% [5–8]. We report here a case of mediastinal thymoma with coexisting axillary pleomorphic sarcoma.

## 2. Case Report

**Figure 1:**
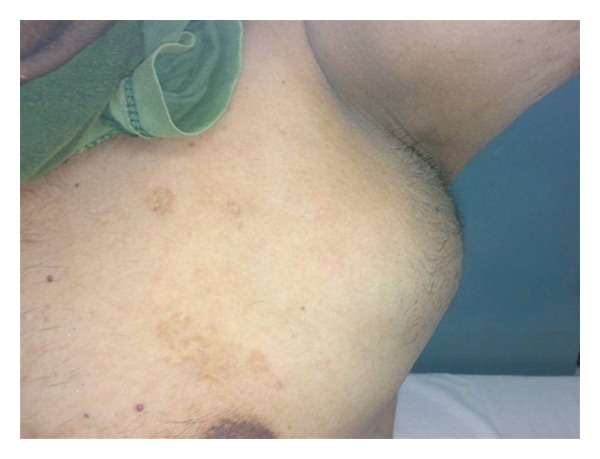
Clinical photograph of patient showing axillary lump.

**Figure 2:**
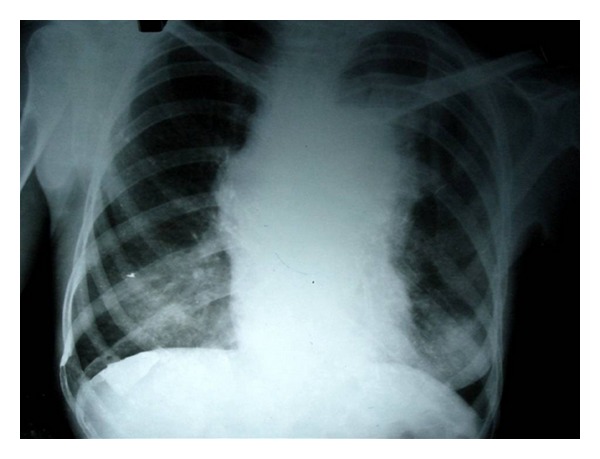
X-ray chest (PA view) showing a mediastinal mass.

**Figure 3:**
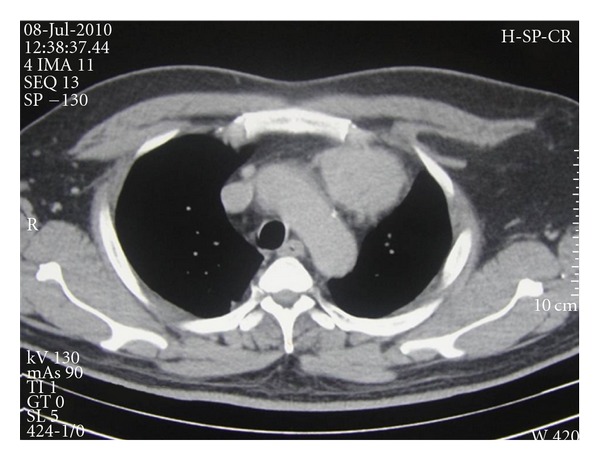
CECT at the level of arch of aorta showing thymic mass.

**Figure 4:**
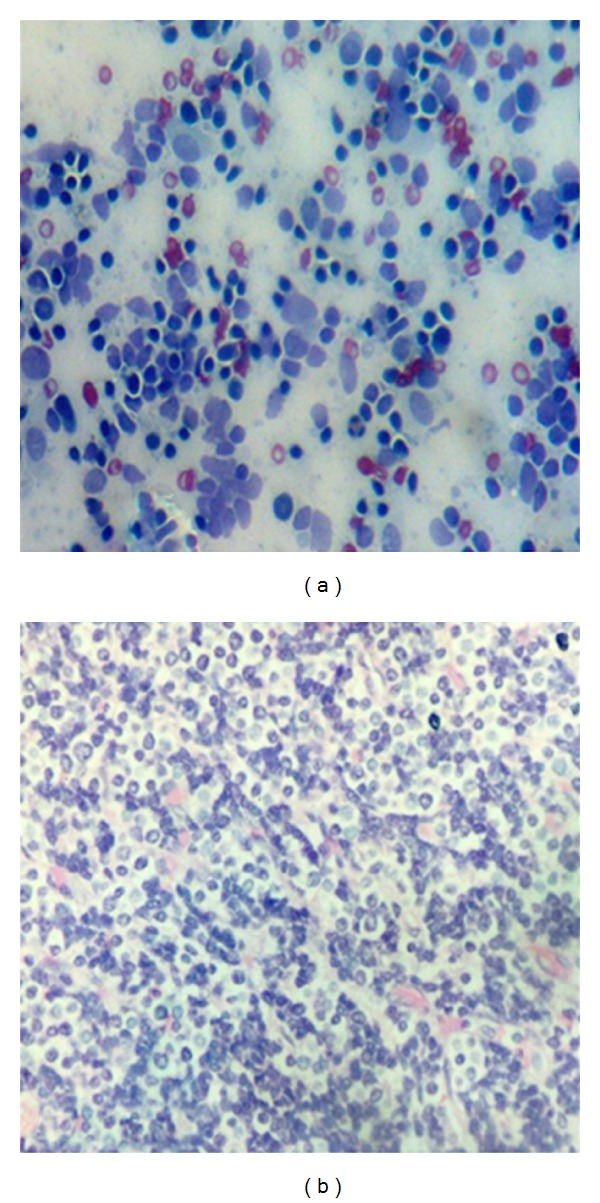
(a) Smears showing dual population of epithelial cells and lymphocytes (H & E ×500). (b) Section showing mature lymphocytes admixed with neoplastic epithelial cells (H & E ×125).

**Figure 5:**
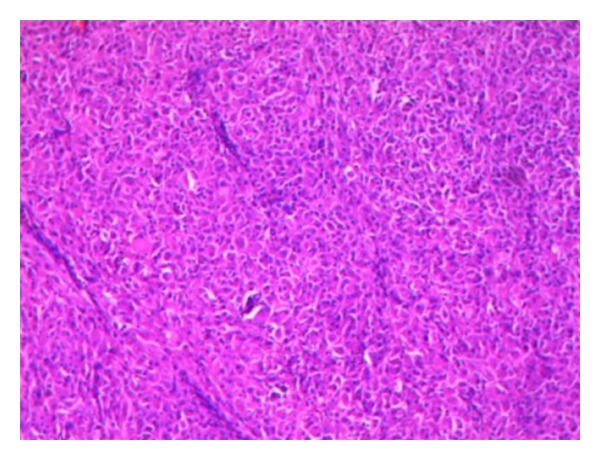
Section showing cells having spindle-shaped pleomorphic and hyperchromatic nuclei (H & E ×50).

**Figure 6:**
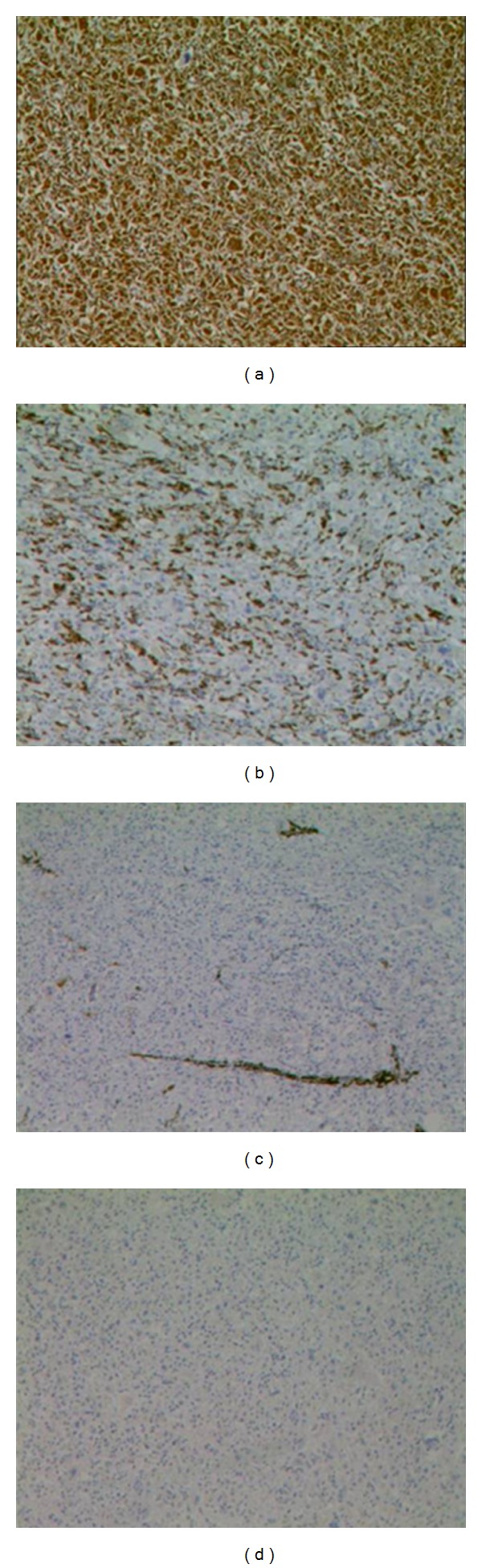
(a) Section showing vimentin positivity ×50. (b) Section showing focal CD 68 positivity ×50. (c) and (d) Sections showing SMA and desmin negativity ×50.

**Figure 7:**
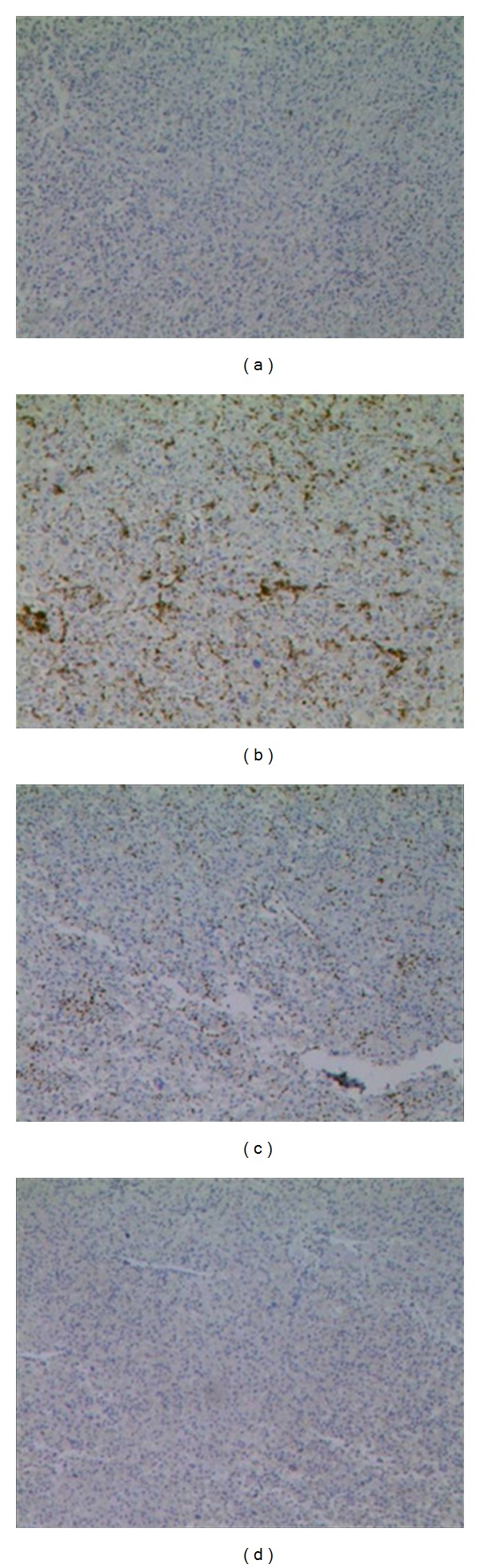
Section showing EMA, CD 45, CD 3, and CD 30 negativity ×50.

A 45-year-old male presented with easy fatigability and lump in the left side of axilla for four months. On general examination mild pallor was noticed. Local examination of left axilla showed a lump of 8 × 8 cm., firm in consistency, fixed to underlying structures, and overlying skin was normal (Figure 1). Investigations showed Hemoglobin—7 gm% and Erythrocyte sedimentation rate—25 mm in first hour. X-ray chest posteroanterior view revealed a mediastinal mass (Figure 2). CECT obtained at the level of arch of aorta and main pulmonary artery showed a well-defined enhancing mass of soft tissue attenuation in the left anterior mediastinum abutting arch of aorta, main and left pulmonary artery with maintained fat planes (Figure 3). Adjacent mediastinal fat plane was partially obliterated. CT guided FNAC was done which showed moderately cellular smears consisting of a dual population of epithelial cells and mature appearing lymphocytes. The epithelial cells comprised of oval to elongated nuclei with dispersed chromatin and inconspicuous nucleoli (Figure 4(a)). Mitosis was not seen. Histopathology of mass revealed loose aggregates of lymphocytes admixed with neoplastic epithelial cells (Figure 4(b)). A diagnosis of thymoma was rendered. Excisional biopsy of axillary mass was done which was composed of cells having pleomorphic, hyperchromatic spindle-shaped nuclei with clumped chromatin (Figure 5). Mitotic rate was high. Occasional multinucleated cell was also seen. Immunohistochemical analysis showed positivity for vimentin (Figure 6(a)) and focal positivity for CD 68 (Figure 6(b)) along with negative smooth muscle actin (Figure 6(c)), desmin (Figure 6(d)), epithelial membrane antigen (Figure 7(a)), CD 45 (Figure 7(b)), CD 3 (Figure 7(c)), and CD 30 (Figure 7(d)). On the basis of the above findings a diagnosis of thymoma with co-existent undifferentiated pleomorphic sarcoma was made. 

## 3. Discussion

The coexisting thymomas with other malignancies is relatively rare, and the most common site for these extrathymic malignancies is colorectal [6, 8] followed by lung, thyroid, prostate, female reproductive organs, breasts, kidney and skin melanoma. Only few cases of co-existent thymoma with coexisting undifferentiated pleomorphic sarcoma not otherwise specified [9, 10] are reported in the literature. The pathogenesis of these association is still unclear. In most of the cases associated tumors were diagnosed either before or at the same time of thymoma as in our case, which suggests that these patients may be genetically predisposed to develop tumors [11]. Several different theories about the pathological basis have been proposed. According to Friedman et al. [12] the onset of thymoma indicates defect within the thymus epithelium which is responsible for immune defect and increased incidence of neoplasia. Another theory suggests the potential ability of thymoma epithelial cells to stimulate T cells which predisposes to the onset of tumors [13, 14]. 

Undifferentiated pleomorphic sarcoma is the most common soft tissue sarcoma in adults, usually arising in extremities with a peak in seventh decade although cases in children have been reported [15, 16]. It can also develop at the site of previous radiation therapy [17]. Enzinger and Weiss classified it histologically into storiform-pleomorphic, myxoid, gaint cell, and inflammatory types [18]. Storiform pleomorphic is the most common histologic type which consists of highly pleomorphic tumor cells arranged in storiform pattern. Immunohistochemistry is required for confirmation of diagnosis. Vimentin positivity and smooth muscle actin, desmin, epithelial membrane antigen, CD 45, CD 30, and CD 3 negativity as in our case made the final diagnosis. Local recurrence and metastasis to distant sites especially lungs and regional lymph nodes are common [19]. Treatment is surgical resection and with or without adjuvant radiation and/or chemotherapy.
